# Effects of Selected Cereal Concentrates and a Gluten-Free Diet on Ovarian, Testicular, and Thyroid Gland Morphology

**DOI:** 10.33549/physiolres.935669

**Published:** 2026-02-01

**Authors:** Peter MAKOVICKÝ, Michaela ŠŤASTNÁ, Lubomír JANDA, Petr JABANDŽIEV, Matěj HRUNKA, Edita JEKLOVÁ, Adam NOREK, Petra STRAKOVÁ, Mária MAKOVICKÁ, Miroslav CHOVANEC, Klaudia KRÁĽOVÁ

**Affiliations:** 1 Department of Infectious Diseases and Preventive Medicine, Veterinary Research Institute, Brno, Czech Republic; 2 Institute of Histology and Embryology, Faculty of Medicine, University of Ostrava, Ostrava-Vitkovice, Czech Republic; 3 Department of Biochemistry, Faculty of Science, Masaryk University, Brno, Czech Republic; 4 Department of Pediatrics, University Hospital Brno, Faculty of Medicine, Masaryk University, Brno, Czech Republic; 5 Cancer Research Institute, Biomedical Research Centre of the Slovak Academy of Sciences, Bratislava, Slovak Republic; 6 Department of Zoology, Faculty of Natural Sciences, Comenius University, Bratislava, Slovak Republic; 7 Institution of Nutrition, Faculty of Nursing and Professional Health Studies, Slovak Medical University in Bratislava, Slovak Republic

**Keywords:** Celiac disease, Gluten, Non-celiac gluten sensitivity, Cereals, Nutrition

## Abstract

Gluten-free diet is currently recommended for people with gluten-related diseases; however some studies document their positive effects also in other diseases. Oppositely gluten is often vilified in nutrition, but serious results about their negative effects in healthy are missing, or controversial. The objective of this study is to compare the effects of different types of diets on ovarian, testicular, and thyroid morphology in an experimental mouse model. Forty-eight (n=48) laboratory mice of the BALB/c line were included in the experiment, divided into 4 groups, and maintained on special diets for 5 weeks. The control group, (6♀, 6♂) was fed a gluten-free diet. The first (E1), second (E2) and third (E3) experimental groups, (6♀, 6♂) were fed a mixture of casein hydrolysate combined with E1: pure extracted gluten in a 30 %:70 % ratio. E2: gliadins at a ratio of 30 %:70 % and E3: avenin at a ratio of 30 %:70 %. At the end of the experiment, the mice were euthanized and ovaries, testes, and thyroid glands were sampled. The samples were fixed in a 10 % formalin solution and processed into hematoxylin-eosin-stained slides. The oocyte and follicle widths of the ovaries were measured; as well as the germinal epithelium and the width of the seminiferous tubules of the testes; as well as the follicle epithelium width and the follicle width of the thyroid gland. The results showed significant differences in the width of oocytes, follicles, testicular seminiferous tubule epithelium, testicular tubules, thyroid follicle epithelium as well as differences in the width of thyroid follicles. Concentrated gluten and gliadin-based diets showed positive results compared to concentrated avenin and gluten-free diets. On the basis of animal experiment using histological methods, it seems that gluten may not be for healthy population harmful and is not recommended to be avoided outside groups of people with gluten-related disorders.

## Introduction

Since the 1990s, the prevalence of celiac disease has gradually increased in the global population of most economically developed countries. This shift marked a significant diagnostic breakthrough, driven by the identification of atypical forms of the disease characterized by broader extraintestinal symptomatology [1]. Some of the research subsequently targeted the identification of triggering mechanisms, and there remain several unanswered questions to this day [2,3]. However, it is evident that this is a genetically determined disease characterized by gluten intolerance with enteritis and villous atrophy of the small intestine [4]. There is less clarity in the interpretation of the pathophysiology of non-celiac gluten sensitivity (NGS), which manifests a broader spectrum of health problems with a focus in the intestinal tract after gluten intake in non-celiac and non-wheat allergic individuals [5,6]. Gluten components of selected cereals are a major cause of pathology in sensitive individuals, but less attention has been paid to the relationship between high-gluten diets and the physiological adaptation of the mucosal relief of the intestinal tract of healthy individuals. It is well known that a portion of the population consciously avoids gluten-containing foods and voluntarily adheres to gluten-free dietary principles, a practice particularly relevant for the treatment of celiac disease and NGS [7–9]. For instance, one study found that a gluten-free diet is among the most popular healthy lifestyle choices globally, with statistics from the USA indicating that one in five individuals adheres to this diet [10]. This trend reflects consumer expectations and, from a financial perspective, presents significant opportunities for manufacturers of gluten-free products [11,12]. It has been experimentally quantified that diets incorporating selected cereals, as well as cereal-free diets, influence small intestinal morphology in rats, specifically affecting the height of intestinal villi and the depth of intestinal crypts [13]. Given its pro-inflammatory properties, gluten has been hypothesized to contribute to the development of both metabolic and neurodegenerative diseases [14,15]. It remains inconclusive whether exposure to higher concentrations of certain cereals induces adaptive changes in the functional organs of healthy individuals. Similarly, it has not been confirmed whether gluten-based diets affect the morphology of other metabolically active organs whose functional changes are involved in several systemic diseases. Some effects between the gluten-free diet and autoimmune thyroid disorders, including reproductive parameters have been previously discussed [16,17]. These questions could be addressed through controlled experiments using laboratory animals subjected to regulated gluten exposure, followed by histological examination of selected organs. The objective of this study is to investigate the effects of concentrated gluten and gluten-free diets on ovarian, testicular, and thyroid morphology using an experimental mouse model.

## Material and Methods

### Ethical aspects

The experiment was approved by the internal ethics committee of the Veterinary Research Institute in Brno, Czech Republic (VRI) under registration number VRI-7/2022 and subsequently approved by the Ministry of Agriculture of the Czech Republic (MZE: 12818/2340). The animal handling complied with the legal directives of the Czech Republic and with the institution’s policy (experiment project number MZe 2340).

### Experimental design

A total of forty-eight (n=48) laboratory mice (♀, ♂) of the BALB/c line were included in the experiment and were reared in the experimental laboratories of VRI from the age of 8 weeks onwards on a diet recommended for the rearing of laboratory animals (Rat/Mouse maintance diet, ssniff, Germany). Mice were obtained from AnLab (Prague, Czech Republic). After a 6-week acclimatization period, mice were divided into 4 groups at 14 weeks of age and further reared on special diets for 5 (40 days) subsequent weeks. The control group (C) (n=12), (6♀, 6♂) was fed a gluten-free diet, which corresponded in composition to the diet used in the acclimatization phase, but with a gluten-free casein hydrolysate protein replacement component (Rat/Mouse maintenance diet, ssniff, Germany). The diet was composed of 3.3 % fat, 19 % protein, 5 % fiber, 35.9 % starch, and 54.6 % non-starch extracts. The first experimental group (E1) (n=12), (6♀, 6♂) was fed a mixture containing casein hydrolysate in combination with pure extracted gluten in a 30 %:70 % ratio. The second experimental group (E2) (n=12), (6♀, 6♂) was similarly fed a mixture containing casein hydrolysate but combined with the addition of gliadins at a ratio of 30 %:70 %. The third experimental group (E3) (n=12), (6♀, 6♂) was also fed a mixture of casein hydrolysate but combined with the addition of avenins at a ratio of 30 %:70 %. In all groups, mice were fed *ad libitum* with free access to drinking water. At the end of the experiment, the mice were euthanized using Isoflurane-based inhalation anesthesia (Isoflurane 1000 mg/g, Werfft, spol. s.r.o., Czech Republic). Subsequently, both ovaries were removed from females, both testes from males, and the thyroid gland was removed from both. After collection, samples were immediately logged, labelled, and fixed in 10 % formalin solution and sent to the histopathology laboratory.

### Sample processing

Samples were fixed for 24 h and processed by a standard histological technique using a histoprocessor (Leica ASP6025, Leica Microsystems, Germany), and then embedded in paraffin blocks using an embedding station (Leica EG1150H, Leica Microsystems, Germany). Serial sections of 2–3 μm thickness were cut on a sledge microtome (Leica RM2255, Leica Microsystems, Germany) and mounted on standard slides (Sussefrost, Medesa, Czech Republic). Sections were stained by transparent hematoxylin-eosin staining (Bammed, Czech Republic). Evaluation was performed with an Olympus BX53 light microscope (Olympus, Tokyo, Japan), which was connected to Zenn-lite analytical software (Zeiss, Germany).

### Sample evaluation

Samples were evaluated diagnostically and morphometrically. In the diagnostic view, individual changes in the organs were evaluated. Additionally, morphometric measurements were performed. Only those follicles were identified on each ovary where the nucleus of the oocyte was also visible, and four separate oocytes and follicles were defined, respecting the histological classification we followed in other experiments [18]. These are primordial follicles, primary follicles, secondary follicles and antral follicles. Individual follicles were selected for each ovary, and the oocyte and follicle width (oocyte + membrana granulosa + thecal layer) were measured using an analytical program. Each oocyte and each follicle were measured crosswise by two measurements. The thickness of the seminiferous tubules and the thickness of the germinal epithelium wall of the seminiferous tubules were measured on the testes samples. A total of 40 seminiferous tubules were selected for each sample. On the thyroid gland samples, the thickness of the follicle epithelium was measured, followed by the thickness of the follicles within 20 thyroid follicles. All measured values are given in μm.

### Statistical evaluation

All statistical analyses were performed in Python (version 3.12) using the packages pandas, scipy, seaborn, and starbars. Data were imported from Excel spreadsheets and grouped according to dietary treatment: E1, E2, E3, and control (C). For each variable, descriptive statistics were first calculated. The distribution of residuals was tested using the Shapiro-Wilk test, which showed that several variables deviated from normality. In addition, Levene’s test indicated that group variances were not always homogeneous and the sample size was unequal. Because of these deviations, the Welch’s *t*-test was used for comparisons. To further reduce the influence of extreme outliers, all datasets were processed with a 5 % symmetric trimming. This means that the lowest and highest 5 % of values within each group were removed before analysis. Trimming ensures that single extreme animals do not disproportionately affect the results while keeping the majority of the data intact. Comparisons were performed only between each diet group (E1, E2, E3) and the control group (C), because of the independent nature of the dietary interventions. Welch’s *t*-tests were conducted separately for each variable. Statistical significance was accepted at p<0.05. Results are reported as raw p-values without correction for multiple testing, because each diet represents an independent intervention and each variable measures a distinct biological structure. In all figures, statistical significance is displayed with stars: p<0.05 (*), p<0.01 (**), p<0.001 (***), and p<0.0001 (****). Non-significant results are indicated as “ns”.

## Results

### Histological findings

**Fig. 1:**
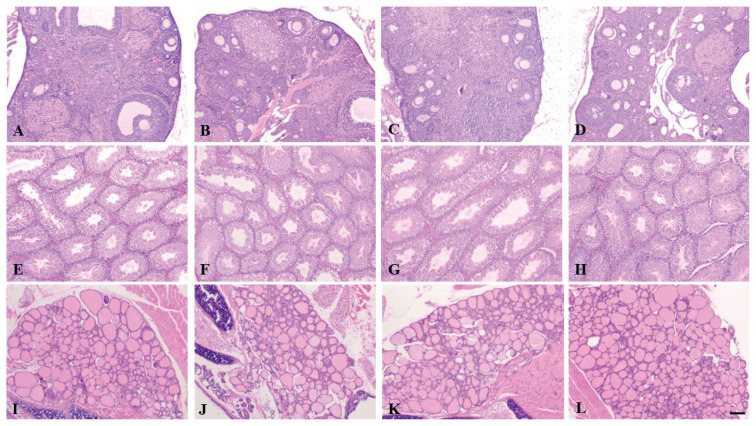
Ovaries with numerous follicles and oocytes present in the cortical region (**A, B, C, D**). Shape similar, ovoid to slightly elongated seminiferous tubules lined with germinal epithelium and sperms in the lumen (**E, F, G, H**). Thyroid follicles with a rim of single-layered epithelium, skeletal muscle fibers and hyaline cartilages of the larynx trapped along the periphery (**I, J, K, L**). In the individual designations, the organs are C-group (**A, E, I**), E1-group (**B, F, J**), E2-group (**C, G, K**), and E3-group (**D, H, L**). All pictures: 40× HE.

All the specimens documented a normal histological picture of the individual organs without pathological changes (Fig. 1).

### Ovarian morphometry

**Fig. 2:**
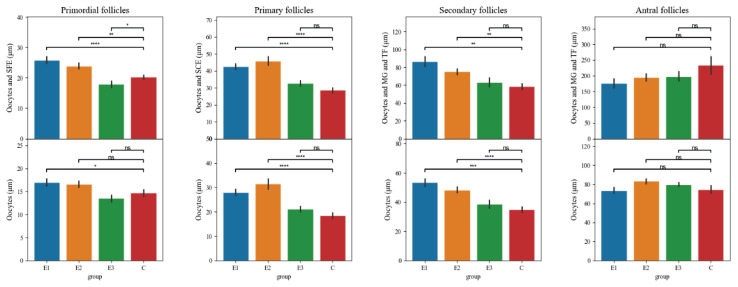
Results of ovarian morphometry. SFE: simple flattened epithelium; SCE: simple cuboid epithelium; MG: membrana granulosa; TF: theca folliculi.

The results are shown in Figure 2. Dietary interventions significantly influenced follicle development in the ovaries. In the first category of primordial oocytes; follicles, the highest values were measured in E1 group (17.01 μm; 25.92 μm), followed by E2 group (16.64 μm; 23.97 μm), then balanced values of groups C (14.73 μm; 20.32 μm) and E3 (13.53 μm; 17.95 μm). For primordial category, all diets showed increases compared with controls (p<0.001 for E1, p<0.001 for E2, p<0.05 for E3). In the category of primary oocytes; follicles, the values were highest in the E2 group (31.53 μm; 45.92 μm), followed by E1 group (28.06 μm; 42.58 μm), then E3 group (21.80 μm; 34.28 μm), and finally C group (18.25 μm; 28.44 μm), with statistically significant differences in diets E1 and E2 which were significantly different from controls (p<0.05 for both), while E3 showed no difference (p>0.3). In the category of secondary oocytes; follicles, the highest values were measured in E1 group (53.47 μm; 86.57 μm), followed by E2 group (48.35 μm; 75.28 μm), followed by E3 group (37.55 μm; 61.57 μm), and the lowest in C group (34.82 μm; 58.73 μm). For secondary category, diets E1 and E2 displayed strong differences from control (p<0.001 for both), whereas E3 showed only borderline significance (p≈0.05). In the category of antral oocytes; follicles, the highest values were measured in C group (74.71 μm; 242.51), followed by E3 group (81.59 μm; 194.04 μm), then E2 group (83.47 μm; 195.24 μm), and finally E1 group (73.76 μm; 176.66 μm). Diets E1 and E2 again showed significant differences (p<0.001 and p<0.01), while E3 did not (p>0.4).

### Testicular morphometry

**Fig. 3:**
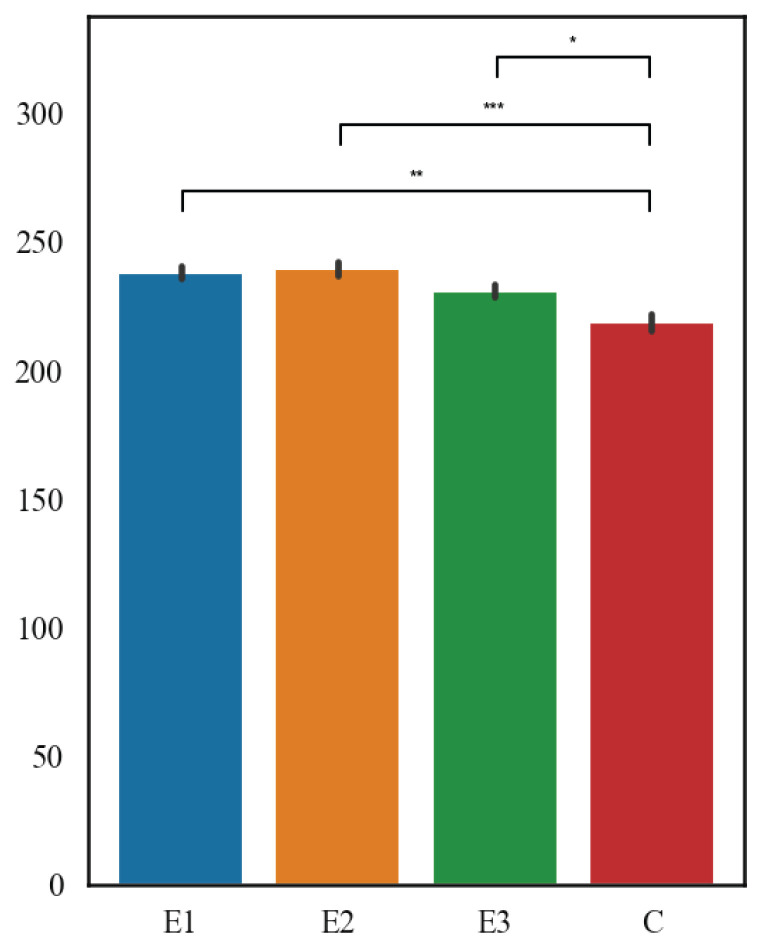
The thickness of the seminiferous tubules.

**Fig. 4:**
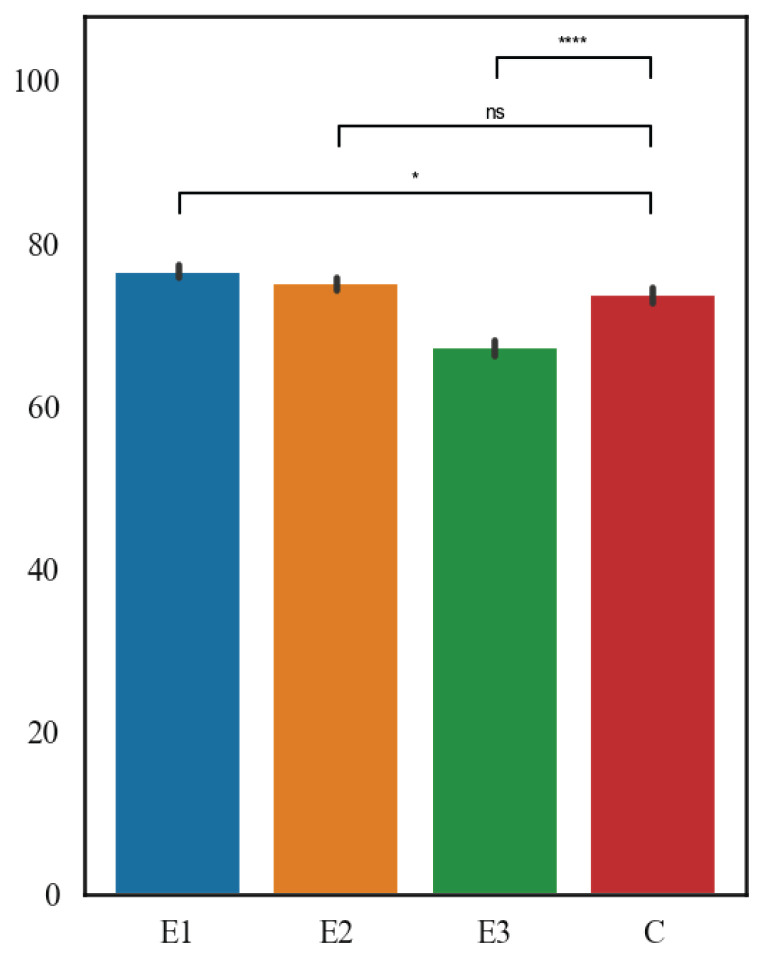
The thickness of the germinal epithelium wall of the seminiferous tubules (μm).

The results are shown in Figures 3 and 4. In terms of the mean thickness of the germinal epithelium of the testicular seminiferous tubules, the highest value was measured in E1 group (76.52 μm), followed by E2 group (75.03 μm), then C group (73.64 μm), and the lowest values were in E3 group (67.16 μm). E1 and E2 diets significantly reduced thickness of germinal epithelium when compared with controls (p<0.01 and p<0.05), whereas E3 showed no effect (p>0.2). Importantly, trimming strengthened the diet E2 effect, revealing significance that was masked in the raw analysis. Within the mean thickness of the seminiferous tubules of the testes, the highest value was measured in E2 group (239.40 μm), followed by E1 group (238.08 μm), then E3 group (230.94 μm), and the lowest values were in the C group (218.72 μm). All diets differed significantly from controls (p<0.001 for E1, p<0.001 for E2, p<0.01 for E3).

### Thyroid gland morphometry

**Fig. 5:**
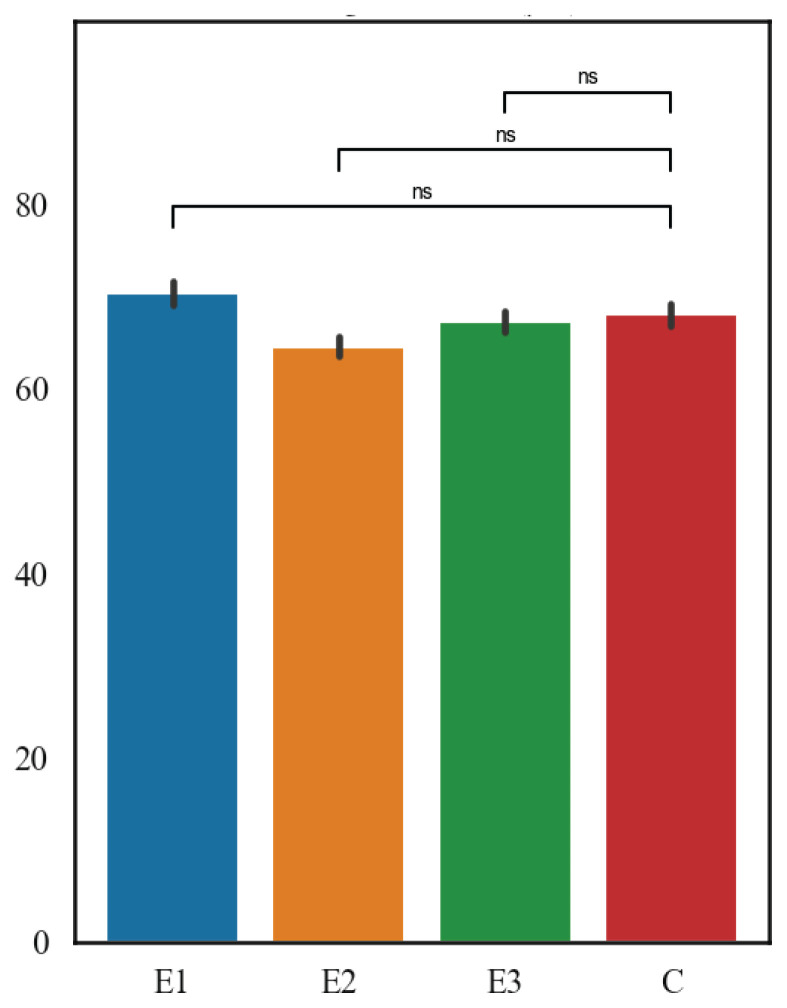
Thyroid follicles average thickness (μm).

**Fig. 6:**
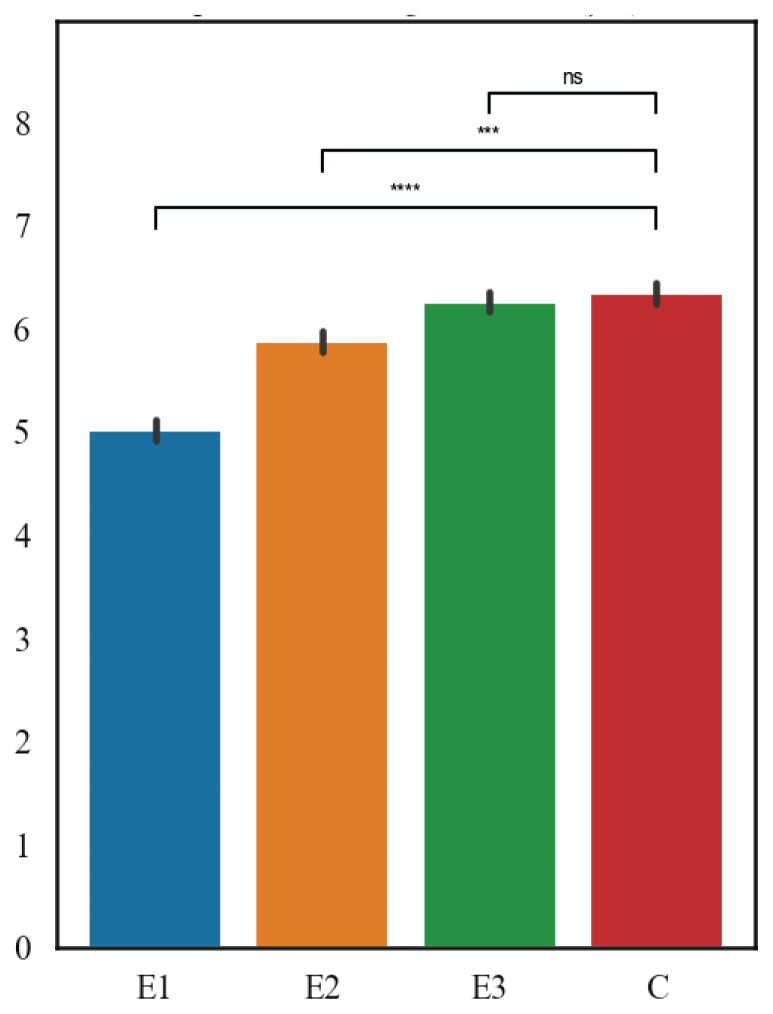
Thyroid gland epithelium average thickness (μm).

The results are shown in Figures 5 and 6. In terms of the mean thickness of the thyroid follicle epithelium, the highest values were measured in C group (6.44 μm), followed by E3 group (6.28 μm), then E2 group (5.8 μm), and finally E1 group (5.01 μm), both E1 and E2 diets showed highly significant differences (p<0.001 for both), whereas E3 showed no effect (p>0.4). Within the mean thickness of thyroid follicles, the highest values were measured in E1 group (70.32 μm), followed by C group (67.57 μm), then E3 group (67.72 μm), and the lowest values were in E2 group (64.57 μm). For follicle thickness, only the E2 diet differed significantly from controls (p<0.05), while E1 and E3 showed no effect (p>0.2).

## Discussion

Healthy nutrition, alongside lifestyle, has been a longstanding focus of attention for both the professional and lay public, with the role of cereals frequently debated. Cereal grains consist of 55–75 % starch, 20 % protein, fat, fiber, ash, and 12–15 % of water [19,20]. The water-insoluble proteins form gluten, which can be separated in 70 % ethanol into an ethanol-soluble fraction (prolamins) and an insoluble fraction (glutenins). In genetically predisposed individuals, gluten peptides interact with HLA-DQ2 and HLA-DQ8 positive immunocompetent cells to initiate a cascade of reactions with the resulting effect of damaging the small intestinal mucosa [21,22]. However, the disease can also manifest extra-intestinally, presenting with a broader range of clinical symptoms in association with other conditions, including reproductive pathology, infertility, or thyroid pathology [23–26]. In healthy individuals, such pathology due to gluten does not occur. However, foods with higher gluten concentrations are known to be more difficult to digest than gluten-free foods, and there are theories in the literature regarding the harmful effects of gluten [27]. The recommendation to avoid gluten is more prominently emphasized in some studies, where it is considered beneficial for individuals with gastrointestinal disorders or as a mean of reducing the risk of cardiovascular diseases [28]. However, the results of other independent studies involving different groups of healthy individuals have found no causal relationship between gluten consumption and the aforementioned pathologies [29,30]. On the contrary, it has been found that it is the purposelessly deployed gluten-free diet that can lead to health problems [31,32]. Thus, it is possible that in the future, diseases arising from long-term adherence to unjustified diets may emerge. On the other hand, the benefits of a gluten-free diet have been documented in relation to diseases whose pathophysiology has not yet been linked to gluten, suggesting that its potential role in the treatment of other conditions should not be overlooked [33–36]. As adult forms of autoimmune diseases are associated with more extensive pathology and later onset, it can be assumed that immunocompetent cells are also in a relationship with cells outside the gastrointestinal tract. This relationship could correspond to a broader, yet undescribed genetic background, which could lead to a more comprehensive classification of the disease in the future [37]. In our study, we investigated the effects of concentrated diets based on gluten components from individual cereals and a gluten-free diet on ovarian, testicular, and thyroid morphology. It remains unclear whether there is a threshold concentration of gluten that can induce changes in the reproductive and endocrine systems of healthy individuals. Additionally, it is unknown whether there are differences in the physiological adaptation of individual organs to the gluten components of selected cereal types. As evidenced by our results, the appearance of the individual organs post-experiment resembled normal structure, with no clear pathological findings. Additional morphometric measurements revealed normal growth of individual oocytes and follicles, with experimental E1 and E2 groups, showing higher mean values of oocyte and follicle width, with statistical significance, when compared with the C group outside the E3 group. This indicates that gluten and gliadins had a positive effect on oogenesis and follicle-genesis. Imik *et al.* [38] compared the effect of soy, corn and wheat gluten on rat ovarian morphology and found no significant differences in morphology, but differences in luteinizing hormone and follicle-stimulating hormone levels were measured in favor of the wheat-gluten group. In our work, we do not have the results of the hormonal profile. In a study comparing the effect of corn and wheat gluten on sperm morphology and characteristics, it was found that high doses of corn gluten had an adverse effect on testicular weight and structure, and on sperm quality [39]. However, our results favor the E1 and E2 groups as beneficial with higher mean values of testicular seminiferous tubule epithelium width and higher mean parameters of testicular seminiferous tubule width, compared with the measured values of the E3 and C groups. These findings may correlate with previously tested hypotheses suggesting that individuals with celiac disease have lower sperm counts in the ejaculate and may be predisposed to infertility, although not all studies have confirmed these conclusions [40,41]. However, it is questionable whether the male reproductive parameters interpreted here are in undiagnosed celiacs or in those diagnosed on a gluten-free diet, and whether these results can also be extended to non-celiac men who are on a gluten-free diet. Reproductive issues are commonly reported as a secondary concern in women with celiac disease. These issues encompass a broad range of problems, including unexplained infertility, complications during pregnancy, delayed menstruation, and early onset of menopause. Conversely, positive outcomes have been documented in women with celiac disease who adhere to a gluten-free diet [42]. However, reproductive parameters are known to be generally under the influence of systemic autoimmune diseases [43]. Looking back, it can be affirmed that distributing the document “Memorandum – Celiac Sprue” to a broader range of professional societies was a prudent decision, as it helped raise awareness of the disease beyond gastroenterology outpatient clinics [44]. Given the described relationship between gluten and autoimmune thyroid inflammation, with some patients reporting positive effects, we also examined thyroid morphology in this study [45,46]. An increased width of the follicular epithelium could indicate higher thyroid activity; however, the measurements do not correlate with the mean follicular width values. Hormonal thyroid profile was not measured. Therefore, we attribute these differences to natural thyroid activity rather than the influence of diets on thyroid morphology. In this paper, we primarily focus on comparing the impact of a gluten-free diet on the morphology of various organs, with several parameters aligning with the effects of oat avenin. This is likely due to avenins being considered one of the most frequently discussed dietary alternatives for celiac patients [47,48]. In clinical practice, upon a diagnosis of celiac disease, children are initially placed on a strict gluten-free diet for an extended period. Once their health status has improved, they are then advised to gradually reintroduce oat products as an alternative to other cereals. This is based on the premise of improving both the variety and the nutritional value of the food, but it is questionable whether this is a universally suitable alternative. The safe daily amount of oats remains a topic of debate [49]. There are variable types of oats on the market, which vary in composition and may be contaminated with the residues of other cereal types [50]. One potential future approach could be to select commercially labelled oat varieties with a proven favorable avenin composition, or for individual producers to test their varieties and market them with such a label. A gluten-free diet is a specialized dietary approach intended for individuals with gluten-related conditions and should not be regarded as universally beneficial for those seeking a healthier lifestyle or improved performance. The ongoing challenge for future research is to explore its potential relevance in the treatment of other diseases, where positive effects and improvements in clinical symptoms have been demonstrated.

## Conclusions

This study examined the effects of concentrated gluten, gliadin, and avenin-based diets, as well as a gluten-free diet, on ovarian, testicular, and thyroid morphology. In an era where anti-gluten sentiment is increasingly widespread, the findings of this study can be relevant both scientifically and socially. Histological analysis revealed no significant pathological changes; however, morphometric analysis identified statistically significant differences in several parameters favoring gluten and gliadin-based diets compared to avenin and gluten-free diets. These differences fall in normal physiological adaptation, documenting cellular activity. A gluten-free diet should be reserved for individuals with specific medical indications, as gluten is not harmful to healthy individuals and should not be intentionally excluded from their daily diet.
